# Ankyloglossia as a Barrier to Breastfeeding: A Literature Review

**DOI:** 10.3390/children10121902

**Published:** 2023-12-08

**Authors:** Eirini Tomara, Maria Dagla, Evangelia Antoniou, Georgios Iatrakis

**Affiliations:** Department of Midwifery, University of West Attica, 12243 Athens, Greece; mariadagla@uniwa.gr (M.D.); lilanton@uniwa.gr (E.A.); giatrakis@uniwa.gr (G.I.)

**Keywords:** ankyloglossia, breastfeeding, breastfeeding difficulties, frenotomy, infant, lingual frenulum, tongue-tied

## Abstract

This is a literature review of ankyloglossia and its correlation with lactation problems. Ankyloglossia, commonly referred to as tongue-tie, brings about functional difficulties and, in some cases, may lead to early weaning. It is crucial to use breastfeeding as the exclusive food source for the first six months of an infant’s life, and the interference of the tongue contributes substantially to success in this regard. Even though there are many publications about ankyloglossia, there are still many controversies about its definition, diagnosis, classification, and treatment decision determined via frenotomy. Some researchers state that the identification of ankyloglossia should be based on morphological and anatomical evidence, while others claim that a short or tight frenulum should be examined in correlation with the impact on the mother–infant dyad during breastfeeding. By encouraging and supporting mothers in coping with feeding difficulties, their lactation experiences are improved, and they can continue breastfeeding.

## 1. Introduction

Nowadays, more and more mothers are becoming aware of the advantages of breastfeeding, and they are choosing breast milk as the fοοd source for their newborns. The global guidance of the World Health Organization (WHO), the American Academy of Pediatrics (AAP), and the European Society for Pediatric Gastroenterology, Hepatology, and Nutrition (ESPGHAN) recommend exclusive breastfeeding for the first 6 months of life and then continuing breastfeeding on demand along with the supplementation of solid foods up to the age of 2 and beyond [1,2,3]. If this recommendation is adopted, it is estimated that approximately 823,000 deaths under the age of 5 will be prevented per year [4]. Based on the global breastfeeding scorecard of 2022, the percentage of babies who breastfed exclusively under the 6 months of age was 48%, while the global target for 2030 is 70% [5].

Alongside the advantages of breast milk, the breastfeeding process itself plays an important role in the stability and stimulation of the perioral muscles of the stomatognathic system, such as the temporal muscle, the masseters, and the orbicularis [6,7]. The growth of the oral cavity muscles, which contributes to lactation, is also a part of the natural training for subsequent mastication [8]. The maturation of the masticatory muscles establishes an effective cycle of breathing and swallowing during lactation.

It is considered that around 90–95% of mothers will be able to breastfeed their babies successfully. As a consequence, mothers set high expectations for breastfeeding and believe that it will be an easy and natural experience [9]. Unfortunately, this discrepancy between expectations and the reality that mothers experience is crucial for the duration of breastfeeding, especially for the 14-day postpartum period [10,11,12]. When lactation problems occur without proper lactational assistance, they may provoke breastfeeding mothers to adopt early weaning and/or formula supplementation [13,14,15,16].

**Figure 1:**
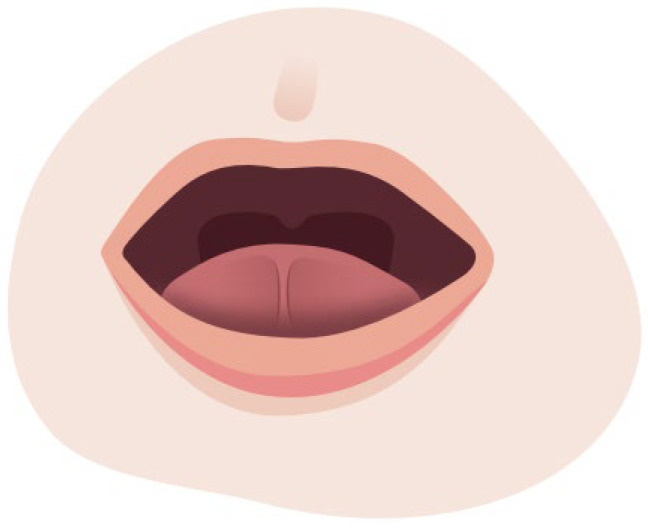
The lingual frenulum of a tongue-tied infant.

A neonatal situation that is linked to the aforementioned lactation problems is ankyloglossia. It is also known as tongue-tie or short lingual frenulum. The purpose of this review is to present broadly accepted criteria for the definition, diagnosis, assessment tools, classification, related problems, and treatment of ankyloglossia, covering the topic in its entirety. For many years, health care providers supporting breastfeeding mothers have been searching for a potential negative impact of a tight or short lingual frenulum on children’s lives, as these issues may reduce their lingual mobility, affect their ability to breastfeed effectively and/or speak clearly in later life, or have an impact on some mechanical and social activities, like licking the lips and maintaining oral hygiene (Figure 1) [17].

The prevalence of tongue-tie among infants varies from 0.1% up to 12%, with the majority of these cases being males [18,19,20,21]. Since ankyloglossia affects many aspects of a child’s life, many professionals from different specialties are involved in consultation and management in clinical practice. These professionals can be, among others, pediatricians, lactation consultants, otolaryngologists, pediatric surgeons, speech therapists, dentists, and orthodontists, and each one approaches tongue-tied infants, near-toddlers, and children from their own professional perspective [22]. This is probably the reason why there are more controversies on this matter and fewer agreements.

## 2. Definition and Diagnosis

**Figure 2:**
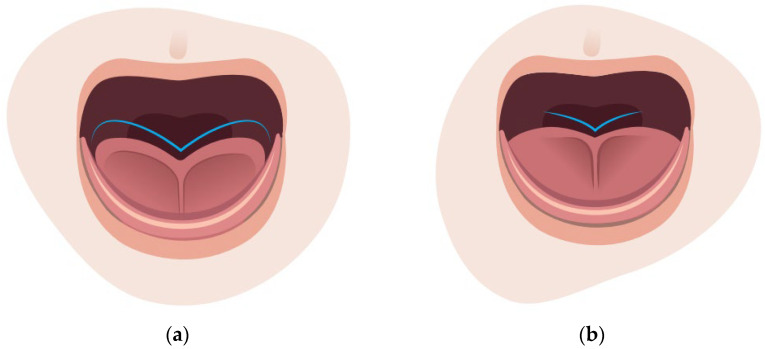
(**a**) Heart-shaped tongue; (**b**) small crevice at the tip of the tongue (V-shaped).

The lingual frenulum is the tissue that connects the undersurface of the tongue with the floor of the mouth [23]. The tongue is developed from the first, second, and third pharyngeal arches over the fourth gestational week [24]. The lingual frenulum is a complicated structure with many layers consisting of dense fibrous connective tissue, a mucous membrane, and fascia or superior fibers of the genioglossus muscle [25,26]. Anatomical variations in these “layers” alter the appearance and mobility of the tongue. Two of the most common clinical features of ankyloglossia are a heart-shaped tongue (Figure 2a) or a small crevice at the tongue tip (Figure 2b) [27,28].

The approaches to addressing lingual frenula that rupture spontaneously have altered over the years, and ankyloglossia is a “fad” condition that seems to be fading away. There are many definitions of ankyloglossia or tongue-tied infants and no consensus on a standard one. Some of them focus on anatomical findings regarding the lingual frenulum, such as its being short, thick, or attached at the tip of the tongue, while others combine the diagnostic criteria with the characteristics of both the mother and infant to describe ankyloglossia. Based on a study by Messner et al. published in 2020, a clinical consensus statement was made, where the authors admitted as a definition that “ankyloglossia is a condition of limited tongue mobility caused by a restrictive lingual frenulum” [17]. The panel of participants consisted of pediatric otolaryngologists who evaluated and treated cases of ankyloglossia in children. They emphasized the tongue’s restricted mobility in correlation with a “restrictive lingual frenulum”. However, whether a restrictive frenulum can be characterized as ankyloglossia without restricted mobility of the tongue is currently under discussion.

Midwives and health care practitioners, who work in clinical practice with breastfeeding mothers daily, should take functional findings into consideration. A detailed lactational history, a clinical examination of a baby’s oral cavity, and close observation of breastfeeding are crucial [17,27,29]. During the clinical examination of an infant’s oral cavity, it is important to assess the tongue’s movement and function in a calm position as well as the shape and the position of movement by allowing the infant to suck the examiner’s gloved finger. In addition, it is essential to palpate the lingual frenulum and observe its elasticity and the length of the tissue and examine the location of the frenulum attachments with respect to the tongue and the floor of the mouth [17]. Last but not least, health care providers should check the range of the tongue’s lifting motion with respect to the palate [30]. Anatomical–functional variations in the length, position, and elasticity of the lingual frenulum may have an impact on an infant’s ability to breastfeed [31].

One of the findings that can be seen during the clinical assessment of an infant’s oral cavity is the ability of the edge of the tongue to protrude out of the mouth [32]. In cases of a tongue with restricted mobility, including the inability to protrude, it is often observed that the tongue is either lying on the floor of the mouth or may be extended up to edge of the gums [33]. Another point is the resting position of the tongue. Normally, the tongue’s position in a state of calmness is on the hard palate. Tongue-tied infants tend to keep their tongues on the floors of their mouths [34]. Similarly, when an infant is crying, the tongue remains on the floor of the mouth, and it may be lifted only at the tip and/or at the side edges [35]. In this case, when there is no mobility limitation, the tongue is lifted straight up. Finally, it is helpful to observe if the tongue can move from side to side after stimulation with the examiner’s finger [36].

## 3. Assessment Tools and Classification

In 1993, the Hazelbaker Assessment Tool for Lingual Frenulum Function (HATLFF) was introduced as the first published tool for ankyloglossia, stemming from the creator’s master’s thesis [37]. It combines five anatomical (appearance of the tongue, elasticity and length of the frenulum, attachment of the lingual frenulum to the tongue, and attachment of the frenulum to the inferior alveolar ridge) and seven functional characteristics (tongue lateralization, lift, and extension; spread of the anterior part of the tongue; cupping; peristalsis; and snapback) of lingual frenulum. Even if it is referred to as a validated tool, unfortunately, there is no public access to these data. Later, in 2015, inspired by Hazelbaker, Ingram et al. created a simple assessment tool named the Bristol Tongue Assessment Tool (BTAT) [38]. It was developed mainly to assess ankyloglossia among infants based on the following four elements: tongue tip appearance, attachment of the frenulum to the lower gum ridge, lift of the tongue with the infant’s mouth wide open (crying), and protrusion of the tongue. Four years later, it was renamed TABBY (Tongue-tie and Breastfed Baby), and twelve pictures were added as box choices [39].

**Figure 3:**
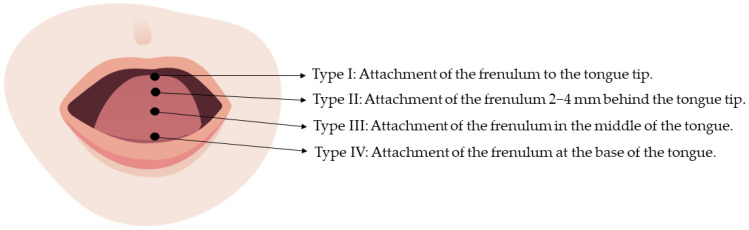
The types based on Coryllos classification [41].

Apart from these assessment tools, there are two more grading tools. Firstly, Kotlow’s classification identifies ankyloglossia according to a single feature: the range of a free tongue. The severity of ankyloglossia is measured in and divided into four classes: Class I—mild ankyloglossia (12–16 mm); Class II—moderate ankyloglossia (8–11 mm); Class III—severe ankyloglossia (3–7 mm); and Class IV—complete ankyloglossia (less than 3 mm) [40]. Another available descriptive classification is Coryllos. The first types, I–II, are referred to as anterior and the other two, III–IV, as posterior ankyloglossia (Figure 3) [41]. Based on the Coryllos classification, emphasis is placed on where the lingual frenulum is tied with the tongue and the floor of the mouth. According to a recent systematic review published in 2022, the HATLFF system and Coryllos classification are used widely [42]. The first one seems superior, since it overcomes Coryllos limitations, because it focuses only on anatomical findings. Consequently, we have no information about the elasticity of the frenulum, its functionality, and whether frenotomy is beneficial [33]. This may be one of the major challenges in ankyloglossia diagnosis. More specifically, a detectable correlation between symptomatic cases of ankyloglossia and validated assessment tools will contribute to determining the “most fitting” cases for the division of the frenulum. Unfortunately, the available scoring systems have not been diligently examined for their validation, and the evidence that guides us most in intervening in tongue-tied infants is the severity of the lactation difficulties experienced by the breastfeeding dyad [43,44,45].

## 4. Lactation Problems

The relationship between ankyloglossia and breastfeeding difficulties hinges on the inadequate mobility of the tongue in forward, upward, and side-to-side motions [45]. Hence, symptomatic tongue-tie will interfere with proper latching onto the breast and, thus, effective sucking with subsequent adequate milk flow into the infant’s mouth, resulting in poor stimulation of the mother’s milk ejection reflex and low weight gain of the infant [46,47,48]. Long feedings, along with poor latching on the infant’s part and a low milk supply and sore or cracked nipples on the mother’s part, are outcomes of this problem. Their main cause is ankyloglossia. For instance, even if we focused on lactation counseling to improve the mother’s low milk supply, resolving milk production effectively would still be uncertain. Even if we manage to accomplish this, the problem could return again. The reason for this is that we have not been tackling the root causes of this problem but instead its symptoms. Health care providers that collaborate with breastfeeding mothers should consider that a thick or tight frenulum may be the cause of their lactational difficulties.

Focusing on mothers, ankyloglossia may affect their breastfeeding experience, milk supply, or breast and nipple anatomy [18]. Therefore, nipple pain and inflicted trauma as well as low milk supply as a result of the ineffective sucking of tongue-tied infants and their difficulty in adequately emptying the breasts may be the reasons that lead mothers to early weaning [18,49]. In fact, intense maternal nipple pain has been reported to be due to persistent difficulty in latching and the subsequent compression of the nipple in the front part of the infants’ mouth between the upper and lower alveolar ridges [45,48]. Walker et al. pointed out that the closer the frenulum is attached to the tip of the tongue, the higher the maternal nipple pain [50]. All these complications result in maternal feelings of stress and failure. Early weaning negatively affects both the infant and the mother because of its psychological implications [12].

When it comes to the infants, some symptoms can be presented, such as long-duration feedings, signs of a lack of satisfaction through feedings, poor or no gain weight, and constant loss of the latch. Meanwhile, supplementary bottle feeding may be used as an alternative approach [18,49]. In a recent cross-sectional study, Campanha et al. confirmed that newborns with ankyloglossia have a 36.07 times higher probability of presenting with lactational problems, especially in their sucking skills [28]. Riskin et al. also emphasized with their findings that tongue-tied infants, regardless of their previously referred to anterior or posterior types of ankyloglossia, will face more breastfeeding difficulties during the first 30 days of life [51].

On the other hand, other studies in the scientific literature, as well as health care providers, contend that ankyloglossia is rarely or never the reason for interfering with feeding, concluding that there is a non-existing correlation between them. As noticed by Messner et al., the professionals involved are mainly pediatricians and otolaryngologists [52]. There is a constant need for further publications, which will emphasize the breastfeeding problems and the types of ankyloglossia [53]. When conservative lactation management is failing and lactational problems still exist, the division of the lingual frenulum can be suggested [49]. Bruney et al. pointed out in a meta-analysis study that frenotomy helps mothers in their lactation experiences by improving their scores on the pain scale and ameliorating lactation problems [54].

## 5. Difficulties with Speech and Solid Foods

A further controversial topic that has been gaining ground is the association between ankyloglossia and speech difficulties. If the tongue has restricted mobility during breastfeeding, could this not lead to future complications in articulation and fluent speech? Only a small percentage of pediatricians admit this correlation exists, while the majority state that it remains unclear [52,55,56]. A recently published systematic review with 1857 participants concluded that there is no correlation between ankyloglossia and speech difficulties. However, the authors claimed that the data were derived from small-sample and low-quality studies [55]. Another relevant study conducted in 2019, which has been marked as the first one to base its cases on tongue-tied children without division, pointed out that the analyzed children had the same speech quality as those treated via frenulectomy [57]. In fact, the data were selected via phone interview according to caregivers’ perceptions, and no objective evaluations of speech and articulation were factored in. Moreover, the diagnosis of ankyloglossia was made according to the ability to protrude the tongue, and none of the available assessment tools or classifications were used. Therefore, it seems that the limitations of the evidence, with a small sample size and heterogeneity in diagnosis, classification, and outcomes, creates a gray zone, limiting the applicability of the published data. High-quality evidence diminishes once popularity invades the field of research.

Following the same theory again, another connection between ankyloglossia and solid foods is considered in [58]. Masticatory function is investigated as one more aspect for attaining a better quality of life. Baxter’s prospective cohort study confirmed this correlation positively. In 37 treated tongue-tied children, progress in their feeding abilities was observed in 83% [59]. Feeding difficulties can occur during an infant’s transition to solid foods and swallowing [60]. In a case study involving a 5-year-old tongue-tied boy, in addition to being a “picky eater”, he demonstrated gagging and vomiting reflexes when eating foods with a variety of textures, but primarily with soft foods [61]. By releasing the tongue, the ability of the tongue to move freely in the oral cavity returns. This also allows food to move freely, and better mastication is accomplished [62].

## 6. Frenotomy

During the last 20 years, in the United States, Canada, and Australia, a rise in ankyloglossia cases has been noticed [43]. However, in European countries like Italy, the Netherlands, and Scandinavian nations, this increase has not been mentioned [63]. Nonetheless, this increase in the cases and divisions of frenulum did not contribute to the universal management of ankyloglossia. The procedure of lingual frenulum division or cutting during infancy is referred to as frenotomy [45]. The available means of division are scissors, a scalpel, and lasers [64]. Frenotomy via laser seems superior since it requires less time and less local anesthetic [65,66]. Furthermore, it facilitates local hemostasis, tissue cauterization, and sterilization [65,66,67]. Nevertheless, using non-thermal techniques of division, less histological tissue injury and inflammation have been reported [68]. In general, complications of the division of the lingual frenulum are quite uncommon [69]. Among the most reported are poor feeding, hemorrhage, inflammation, and trauma inflicted on the local tissues in the oral cavity [64].

The data provided in the literature regarding the optimal time for the incision are quite scarce [70]. However, when there is a case with a tongue-tied infant with breastfeeding difficulties and conservative management fails, the lingual frenulum should be divided as soon as possible [71]. If the case is a tongue-tied infant without feeding problems, we should follow up with lactation consulting, and division may be offered, if and when it is needed, based on the subsequent challenges regarding solid foods and speech. Finally, if the mother is encountering breastfeeding problems and her tongue-tied infant has no complications, then, firstly, we may follow up with lactation consultation, and then if the complaints of the mother persist, we may discuss surgical intervention [53].

In addition, for the first aforementioned case with the symptomatic tongue-tied infant, the clinical consensus statement of Messner et al. is also in favor of an early frenotomy during the first month of life [17]. An interesting issue about frenotomy is parental perception. In 2019, Caloway and her colleagues offered a multidisciplinary evaluation with lactation consulting of feeding for 115 patients before performing a frenotomy. After helping the mothers based on their breastfeeding difficulties, more than half of these cases (62.6%) did not proceed in undergoing a division of the frenulum [72]. Both health care providers and parents should be informed in advance about the advantages, disadvantages, and possible complications of frenotomy.

A Cochrane review verified that frenotomy eliminates mothers’ nipple pain in the short term [73]. Three more studies confirmed this statement using statistically significant results [74,75,76]. Ghaheri et al. confirmed, in a prospective cohort study, that the division of the frenulum is associated with improved lactation outcomes, starting from one week after the division to up to one month [75]. In another study, mothers reported a reduction in nipple pain ranging up to 92% after 3 months of frenotomy [77]. There are also studies that assess mothers’ feelings and willingness to continue to breastfeed their infants as a positive outcome of the division [70,75,78]. On the other hand, when it comes to the infant, it has been noticed that feedings do not take as much time and that there are fewer feedings during the day, with better latching and improved milk transfer [79,80]. Similarly, Miranda and Milroy indicated that there was an improvement in neonatal growth 14 days after the division, as determined via weight gain centiles [81].

Once a frenotomy is performed, there is some recommended advice and there are some interventions that can aid in the healing process and eliminate the rates of the regeneration of the tissue [82]. Firstly, it is helpful for an infant to breastfeed immediately after frenotomy, due to hemorrhage prevention [83]. Secondly, there are post-procedure exercises, which are performed by the parents, in which thoroughly clean hands or gloves are used. After the division of the frenulum, those who assist in lactational counseling should educate parents on how to massage the division’s spot by adding some pressure [84]. Also, it is recommended to stimulate the infant’s tongue using lifting movements directed toward the palate and from side to side. Therefore, the aforementioned myofunctional exercises will enhance the functional mobility of an infant’s tongue by revealing its new range of motion. The frequency of the exercises is four to six times during the day [75,85]. Last but not least, it would be useful to arrange a post-frenotomy meeting with the mother in order to reassess the lactation difficulties and the progress of the breastfeeding dyad.

## 7. Conclusions

In conclusion, the available published data are divided and controversial regarding the diagnosis, classification, management, and treatment of ankyloglossia, enhancing the importance of the relevant training, education, and experience for the professionals who support breastfeeding mothers and tongue-tied infants. Focusing on definition and diagnosis, it would be a good starting point to stop excluding functional findings from definitions since we keep taking them into consideration to diagnose and classify ankyloglossia. As Ms. Watson mentioned, “classifications should be correlated with function to be meaningful” [33]. It seems that it would be helpful for health care providers who are active in consultation regarding breastfeeding and ankyloglossia to combine anatomical and functional findings to define and diagnose ankyloglossia during the first days or months of an infant’s life.

Many authors claim that the creation of a standard protocol or a validated assessment tool for ankyloglossia as a diagnostic instrument in clinical practice would be effective in diagnosis, even if the currently existing tools have not yet managed to overcome this difficulty in diagnosis. It would be more beneficial to use the aforementioned findings in simple forms, taking them into account with respect to the history and examination of the oral cavity instead of using them as tools with scores. It is known that the more delayed a diagnosis, the more likely it is that the association between symptomatic tongue-tied infants with the abandonment of breast feeding will be observed. Time remains a critical confirmed dimension for treatment and surgical intervention regarding tongue-tied infants. Since there is a lack of universal guidelines on the diagnosis, management, and treatment of ankyloglossia, a consensus statement could create a new era of collaboration, with more health care providers aligned in the future. For instance, a consensus on tongue-tied infants and breastfeeding reached by professionals who work in lactation consulting could be effective.

In Greece, as midwives, we do not make the diagnosis or intervene with division in cases of ankyloglossia, but we can play a key role in the detection and referral of these cases. It is important to listen to mothers, observe the breastfeeding dyad, examine the infant’s oral cavity, and inform them of and refer them to a specialist to re-examine the infant and intervene surgically, if needed. By examining an infant’s oral cavity, we acquire more and more experience regarding the variety of the tissue by using palpation, and we can combine these findings and characteristics with maternal complaints about breastfeeding. It is essential to invest more in future training regarding tongue-tied infants in midwifery and to collaborate with pediatricians, otolaryngologists, and pediatric surgeons in order to offer a multidisciplinary and individualized approach to assessing the breastfeeding dyad.

## Data Availability

Not applicable.
